# Preparation and Optimization of O/W Emulsions Stabilized by Triglycerol Monolaurate for Curcumin Encapsulation

**DOI:** 10.3390/molecules27248861

**Published:** 2022-12-13

**Authors:** Guiqiong Zhang, Qiang Zhang, Lan Wang, Lei Ji, Pengbing Han, Fengju Zhao, Qun Su

**Affiliations:** 1 Gansu Provincial Cancer Hospital, Lanzhou 730050, China; 2 Gansu Provincial Academic Institute for Medical Research, Lanzhou 730050, China

**Keywords:** curcumin, O/W emulsion, triglycerol monolaurate, encapsulation

## Abstract

Curcumin is one of the most studied chemo-preventive agents, which may cause suppression, retardation, or inversion of carcinogenesis. But its application is currently limited because of its poor water-solubility and bioaccessibility. A curcumin O/W emulsion was prepared by high-pressure homogenization, using triglyceride monolaurate as an emulsifier and medium chain triglycerides (MCT) as the oil phase. The effects of emulsifiers, emulsifier concentration, oil type, oil-to-water ratio, and homogenization pressure and processing cycles on the physical stability and droplet size distribution of curcumin-encapsulated O/W emulsions were evaluated in this study. The results showed that the mean droplet size of the O/W emulsions remained remarkably stable during 60 days of storage under both light and dark conditions. Curcumin retentions in O/W emulsions after 60 days of storage under light and dark conditions were 97.9% and 81.6%, respectively. In addition, during the simulated gastrointestinal digestion process, the mean droplet size of the O/W emulsions increased from 260 nm to 2743 nm after incubation with simulated gastric fluid (SGF) for 24 h, while the mean droplet size remained unchanged after incubation with simulated intestinal fluid (SIF). The results displayed negligible changes in curcumin content during incubation with simulated gastrointestinal fluids, indicating that effective protection of curcumin was achieved by encapsulation in the O/W emulsion. It is expected that curcumin will acquire high bioaccessibility and bioavailability when the O/W emulsion is to be used in clinical applications.

## 1. Introduction

Curcumin is a natural compound extracted from the rhizome of turmeric (Curcuma longa), a natural plant that has had known medicinal uses for more than 4000 years. Curcumin is of great interest for its therapeutic activities, such as antioxidant and anti-inflammatory effects [1,2]. Therefore, it is widely used for arthritis, liver and neurodegenerative diseases, obesity, and various cancers [3,4,5,6]. In comparison to classical chemotherapy, chemoprevention is a promising anti-cancer approach with fewer side effects. Curcumin is one of the most studied chemo-preventive agents, which may cause suppression, retardation, or inversion of carcinogenesis [7]. However, the clinical application of curcumin is often challenging due to its high hydrophobicity and poor water solubility, resulting in low bioavailability due to its poor absorption into plasma and the target tissues [8]. The low bioavailability of curcumin is also attributed to its fast metabolism and short half-life. In addition, curcumin is photosensitive with limited chemical stability during manufacturing and storage.

To overcome these challenges, several types of encapsulation nanotechnologies, such as liposomes [9,10], emulsion-based systems [11], nanoparticles [12,13], hydrogels [14,15], and many others have been applied to improve the curcumin solubility, stability, and bioavailability [16,17]. Emulsion-based systems have attracted the most research attention, as they are easy to prepare, low-cost, have high encapsulation capacity, and are characterized by manipulatable size, structure, and properties. Curcumin is encapsulated in small lipid droplets in O/W emulsions, thereby improving its water dispersibility and chemical stability [18]. The ability of an emulsion to effectively encapsulate curcumin and improve its stability, bioaccessibility, and bioavailability depends on its droplet size, oil composition, volume fraction, dispersion conditions of curcumin in the oil phase and the type of interfacial materials [11]. It has been reported that the bioaccessibility of curcumin encapsulated in O/W emulsions depends on the chain length of the lipid phase, with medium-chain triglycerides giving a higher encapsulation than short- or long-chain triglycerides [19]. It is also evidenced by the enhanced anti-inflammation activity of curcumin encapsulated in O/W emulsions. This anti-inflammatory activity was further enhanced when the emulsion droplet sizes were reduced to below 100 nm [20]. Consequently, it is essential to optimize these parameters in order to develop effective delivery systems for curcumin.

The current study aimed to examine the impact of some key factors on the formation and stability of curcumin-loaded O/W emulsions to protect curcumin during storage and in vitro gastrointestinal digestion.

## 2. Results and Discussion

### 2.1. Optimization of O/W Emulsions

O/W emulsions were prepared with high-speed dispersion combined with high-pressure homogenization, followed by stability analysis and droplet size measurement. The Turbiscan Stability Index (TSI) and size distribution were applied to evaluate the properties of the O/W emulsions. The TSI value combines all physical instabilities of the sample, including creaming, sedimentation, coalescence, flocculation, Ostwald Ripening, etc. [21]. It gives a number for evaluating the stability of the sample, which reflects the degree of instability of the tested sample. The larger the TSI, the more unstable the sample. According to Stokes’ law, smaller droplets can decrease the velocity of gravitational separation further to increase the stability of emulsions.

#### 2.1.1. The Type of Emulsifiers

**Figure 1:**
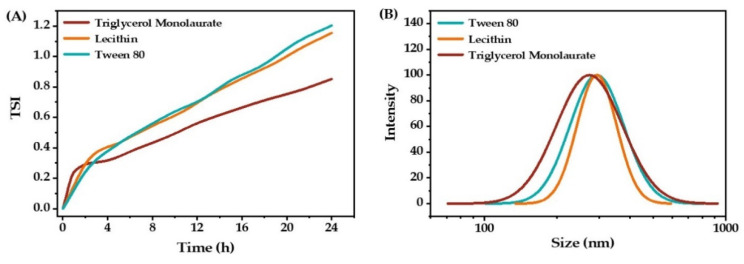
The stability (**A**) and droplet size distribution (**B**) of O/W emulsions stabilized by triglycerol monolaurate, lecithin, and Tween 80.

The stability of an emulsion is closely related to the emulsifier [22]. In this study, triglycerol monolaurate, lecithin, and Tween 80 were used as emulsifiers to prepare O/W emulsions. The stability and droplet size distribution of emulsions using these three emulsifiers are shown in Figure 1.

After 24 h test, the minimum TSI value (0.85) was observed when triglycerol monolaurate was used as the emulsifier (Figure 1A). When lecithin and Tween 80 were used as emulsifiers, the TSI values were 1.15 and 1.20, respectively. In addition, the mean droplet size of the emulsion stabilized by triglycerol monolaurate was also a little smaller than the emulsions stabilized by lecithin and Tween 80. These results show that the most stable emulsion was obtained when triglycerol monolaurate was used as the emulsifier.

#### 2.1.2. Concentration of Triglycerol Monolaurate

**Figure 2:**
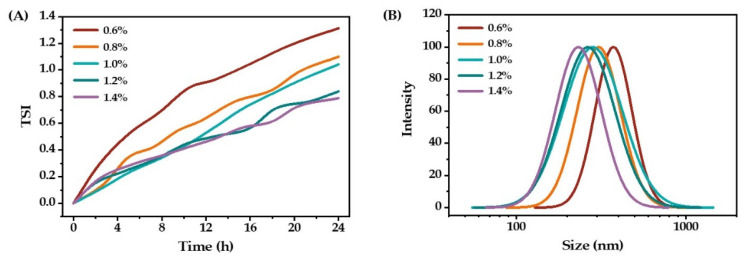
The stability (**A**) and droplet size distribution (**B**) of O/W emulsions prepared with different concentrations of triglycerol monolaurate.

Figure 2 shows the stability and droplet size distribution of emulsions prepared with different emulsifier concentrations. As the triglycerol monolaurate concentration increased, the emulsion’s TSI value decreased. When the concentration of triglycerol monolaurate was 1.2%, the TSI value of the emulsion was about 0.80. However, once the emulsifier concentration was above 1.2%, the TSI value barely changed. As expected, increasing the emulsifier concentration resulted in a decrease in droplet size (Figure 2B). This is because the small droplet size meant a larger surface area, requiring more emulsifiers [23]. From the viewpoint of stability and economic efficiency, the optimal mass concentration of triglycerol monolaurate was 1.2% for the preparation of O/W emulsions.

#### 2.1.3. Oil Type

**Figure 3:**
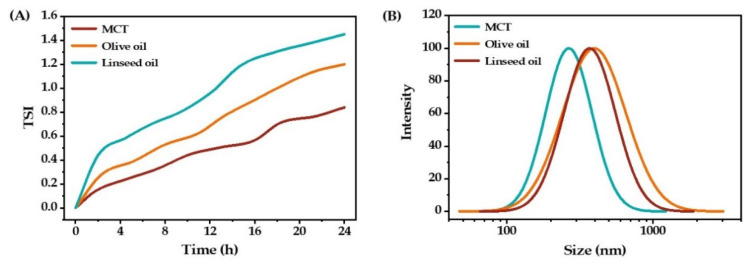
The stability (**A**) and droplet size distribution (**B**) of O/W emulsions with different oil types.

The oil phase is one of the crucial components of the emulsion, and its composition and properties affect the stability and droplet size of the emulsion [19]. Medium-chain triglycerides (MCT), olive oil, and linseed oil were applied as the oil phase for preparing O/W emulsions. The stability and droplet size distribution of the prepared emulsions are shown in Figure 3. The smallest TSI value and mean droplet size were found when MCT was used as the oil phase, which means the most stable emulsion was obtained. Moreover, MCT is known to be the desired solvent for curcumin regardless of the dissolution treatments [22].

#### 2.1.4. Oil-to-Water Ratio

**Figure 4:**
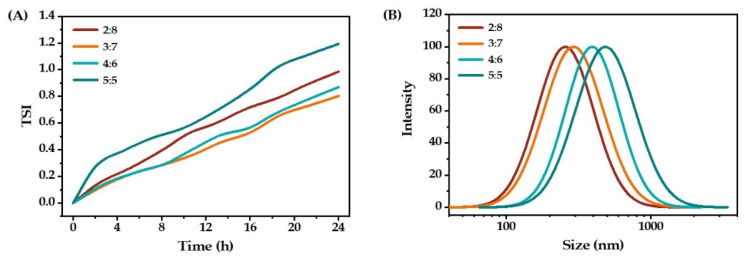
The stability (**A**) and droplet size distribution (**B**) of O/W emulsions prepared with different oil-to-water ratios.

It is generally desirable to have a high content of the oil phase in the O/W emulsion, as this allows the loading of more hydrophobic active ingredients. However, it is known that the oil content can also affect the droplet size of the emulsions through the phenomenon of coalescence. Thus, the oil-to-water ratio is an important parameter in evaluating the stability of O/W emulsions produced [24]. With an increase in oil content, the TSI value first decreased and then increased. When the oil-to-water ratio was 3:7, the smallest TSI value was observed. As seen in Figure 4B, the mean droplet size of the emulsion increased with the increasing oil content. In this case, the excessive oil could not be covered by the emulsifier, which would affect the droplet size of the emulsion. Based on the TSI value and particle size results, the oil-to-water ratio of 3:7 was selected for the next experiment.

#### 2.1.5. Homogenization Pressure and Processing Cycles

When prepared by the high-pressure homogenization method, the droplet size of the coarse emulsion particles decreases gradually under the action of shear, collision, and cavitation effects, giving more stable emulsions. The pressure and processing cycles of the high-pressure homogenization significantly affect the droplet size of the emulsion [24].

**Figure 5:**
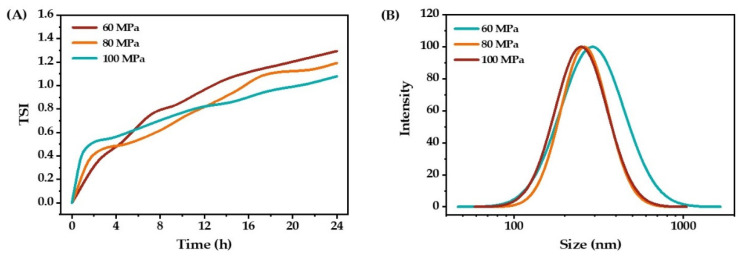
The stability (**A**) and droplet size distribution (**B**) of O/W emulsions prepared under different homogenization pressure.

The effect of homogenization pressure on emulsion properties is mainly manifested in shear and turbulence, which are pressure-dependent. As shown in Figure 5A, the TSI value decreased with the increase in homogenization pressure, indicating that the emulsion stability increased with the increase in homogenization pressure. It can be seen from Figure 5B that the larger the homogenization pressure, the smaller the particle size. However, the droplet size distribution showed no significant difference when the homogenization pressure was 80 MPa and 100 MPa. Considering energy consumption, the homogenization pressure of 80 MPa was selected for the next experiment.

**Figure 6:**
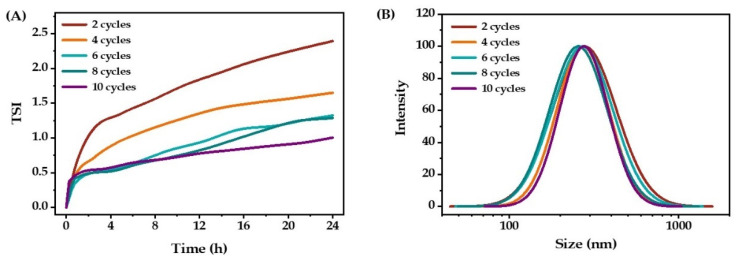
The stability (**A**) and droplet size distribution (**B**) of O/W emulsions prepared after different homogenization processing cycles.

Increasing the number of homogenization processing passes resulted in a significant decrease in TSI values (Figure 6A), which indicated that increasing the processing cycles can improve homogenization efficiency and give more stable emulsions. This may be due to the inhomogeneous energy generated by the homogenizing valve, resulting in the wide size distribution of emulsion; thus, multiple homogenization processing cycles are necessary for preparing more stable emulsions. 

It can be seen from Figure 6B that with the increase in the number of homogenization cycles, the mean droplet size of the emulsion gradually decreased, but when increased to 10 cycles, the mean droplet size of the emulsion increased. This could be caused by the aggregation of small particles, as the increased surface area and the interaction between particles [25]. Based on the results of TSI value and particle size, six or eight processing cycles were the optimal condition for preparing the emulsion.

#### 2.1.6. Multivariate Optimization by Response Surface Methodology (RSM)

Based on the univariate optimization experiments, the effect of four parameters on the stability of O/W emulsions was studied using the RSM method according to Box–Behnken design (BBD). A 4-factor-3-level design was undertaken using Design-Expert 12 software, and the experimental data are shown in Table 1.

The results were confirmed by statistical analysis and a second-order equation was obtained for the stability of the emulsions (quantified by TSI value):

Y = 0.744 − 0.04X_1_ + 0.0208X_2_ − 0.0233X_3_ − 0.0075X_4_ + 0.0125X_1_X_2_ − 0.015X_1_X_3_ + 0.0025X_1_X_4_ + 0.0425X_2_X_3_ − 0.0475X_2_X_4_ − 0.0175X_3_X_4_ + 0.01634X_1_^2^ + 0.0822X_2_^2^ + 0.0684X_3_^2^ + 0.0922X_4_^2^ (1)

where Y is the TSI value after 24 h-test, while X_1_, X_2_, X_3,_ and X_4_ refer to the concentration of triglycerol monolaurate (wt%), the oil phase fraction (%), homogenization pressure (MPa), and processing cycles (number), respectively. 

The coefficient of determination (R^2^) and adjusted R² of the model were 0.9683 and 0.9366, respectively. ANOVA was employed to verify the importance of the model. As the calculated *p*-value (<0.0001) for the model (row #1 in Table 2) is lower than the chosen value of alfa (0.05), we reject the null hypothesis and accept the alternative hypothesis that the model describes properly the observed data. The model shows no “Lack of Fit” as seen by the *p*-value of 0.4536, larger than 0.05. The linear coefficients (X_1_, X_2_, and X_3_), quadratic term coefficients (X_1_^2^, X_2_^2^, X_3_^2^, and X_4_^2^) and interaction coefficients (X_2_X_3_ and X_2_X_4_) were significant (*p*-value < 0.05). The other term coefficients were not significant (*p*-value > 0.05). Among the four independent factors, the concentration of triglycerol monolaurate (X_1_) showed the most significant effect on emulsion stability, followed by homogenization pressure (X_3_). The interactive effects between oil phase fraction (X_2_) and processing cycles (X_4_), and oil phase fraction (X_2_) and homogenization pressure (X_3_) were significant.

In this study, the optimal conditions of the variables were investigated for achieving a minimum TSI value, implying the highest stability of O/W emulsions. A minimal TSI value of 0.72 was predicted by the model with a set of reaction conditions suggested: triglycerol monolaurate concentration of 1.23 wt%, oil phase fraction of 28.1%, homogenization pressure of 85.0 MPa, and eight processing cycles. Under these conditions, three experiments were conducted and an average TSI value of 0.73 (±0.08) was obtained, which is reasonably close to the predicted value. This further confirms the validity and adequacy of the model.

### 2.2. Characterization of O/W Emulsions

**Figure 7:**
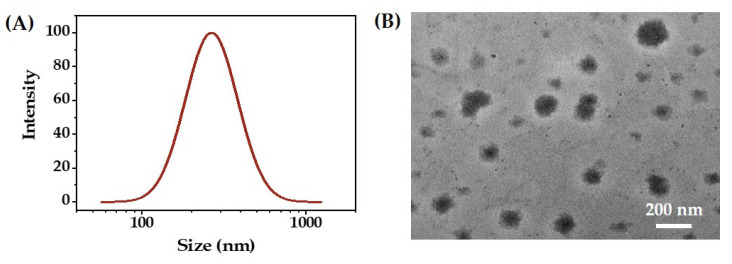
The droplet size distribution (**A**) and TEM image (**B**) of the curcumin O/W emulsion.

Under the above-optimized conditions, an O/W emulsion was prepared using triglycerol monolaurate with a mass concentration of 1.23%, MCT as the oil phase with an oil fraction of 28.1%, under 85.0 MPa of homogenization pressure, and with eight processing cycles. The droplet size distribution of the resulting O/W emulsion is presented in Figure 7A, which shows that the mean droplet size was about 260 nm. 

Figure 7B shows the TEM image of the O/W emulsion. The droplets deviated markedly from a spherical shape, similar to those reported in the literature [26]. This phenomenon may be due to the morphological change during the drying process for the sample preparation. The mean droplet size in the TEM image was slightly smaller than that measured by the DLS method.

### 2.3. Storage Stability of O/W Emulsions

The stability of nanoparticle dispersions is essential to evaluate their feasibility in delivery systems. In order to investigate the physical stability of the optimized O/W emulsion, the change in the mean droplet size and PDI value against storage time were studied by the DLS method.

**Figure 8:**
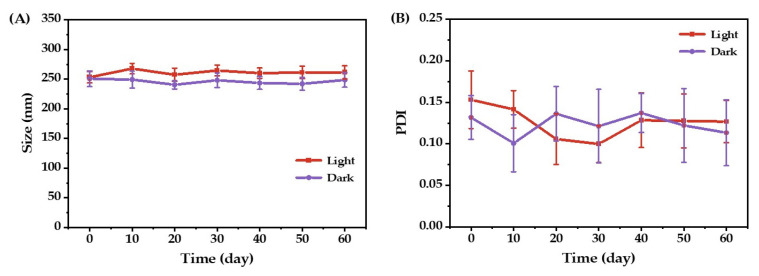
Mean droplet size (**A**) and PDI (**B**) of the optimized O/W emulsion as a function of storage time.

The curcumin-loaded emulsion was stored at room temperature for 60 days under both natural light (light) and dark conditions (dark). The mean droplet size and PDI value were determined at regular intervals, and the results are displayed in Figure 8. The mean droplet size of the curcumin-loaded emulsion under both light and dark conditions remained remarkably stable during the tested 60 days. The PDI value fluctuated, but not markedly, remaining under 0.2, exhibiting the outstanding physical stability of the O/W emulsions prepared in the present study. The presence of emulsifiers impedes small-size droplets from aggregating into larger ones and gravitational separation, thereby producing stable emulsions. Similar results have been reported for nano-emulsion using Tween 80 as an emulsifier [22].

**Figure 9:**
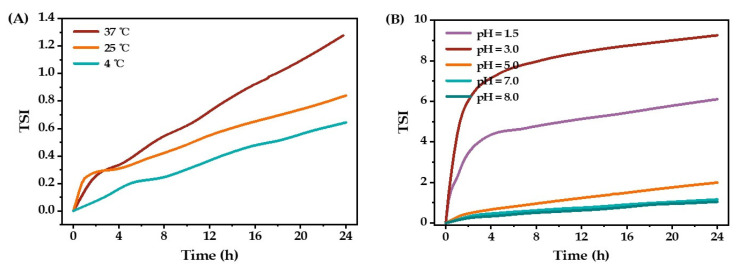
The stability of the optimized O/W emulsion under different temperatures (**A**) and pH (**B**).

In addition, the influence of storage temperature and pH on the stability of the curcumin O/W emulsions is shown in Figure 9. The stability of the curcumin O/W emulsions significantly decreased as the storage temperature increased (Figure 9A). The emulsion system is thermodynamically unstable. Temperature plays an important role in the thermodynamic movement. The molecular thermal motion rate increases with the increase in temperature, leading to instability. Thus, storage at a low temperature is more beneficial to the stability of the emulsion. The influence of pH on the stability of curcumin O/W emulsions was also significant (Figure 9B). The curcumin O/W emulsion showed the best stability under neutral, weakly acidic, and weakly alkaline conditions. Under acidic conditions, the emulsion became unstable, especially at pH 3.0.

### 2.4. Storage Stability of Curcumin Encapsulated in the O/W Emulsions

Curcumin is highly hydrophobic and sensitive to the external environment [16]. When encapsulated in the hydrophobic core of an O/W emulsion, the solubility was markedly increased, and the stability can also be notably improved [11,27]. In the present study, the stability of the encapsulated curcumin was evaluated. The concentration of curcumin encapsulated in the O/W emulsions was measured as a function of storage time. The emulsions were stored at room temperature for 60 days under both natural light (light) and dark conditions (dark).

**Figure 10:**
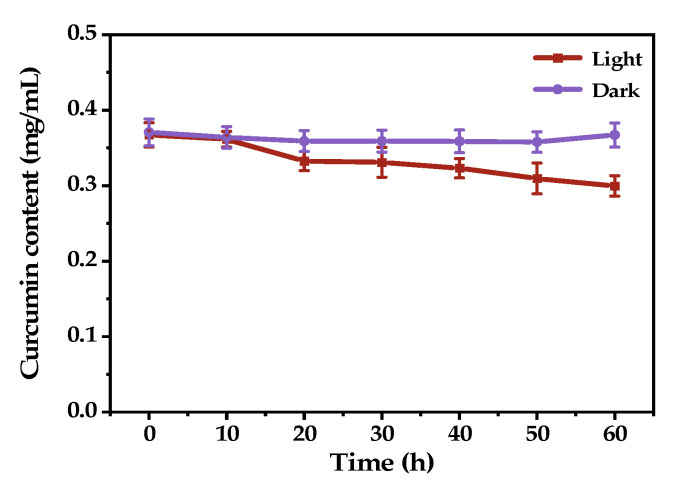
Curcumin content variation as a function of storage time.

Figure 10 shows the change in curcumin concentration in the O/W emulsions as a function of time. It was found that the curcumin concentration in the O/W emulsion stored in the dark condition remained almost unchanged during storage, and the retention of curcumin was 97.9 ± 0.9% after 60 days. These results indicated that curcumin’s stability was remarkably improved by incorporating it into the oil droplets of the O/W emulsion. When exposed to natural light, the curcumin concentration in O/W emulsion decreased gradually as a function of time, and 81.6 ± 0.4% of curcumin remained after 60 days. The retention of curcumin in dark conditions was significantly higher than in natural light because light irradiation promotes curcumin degradation.

Overall, the desired protection of curcumin was obtained in the present study compared to the results of prior studies focused on the encapsulation of curcumin. In one study, after 60 days, 70% of the initial amount of curcumin remained in the nano-emulsion using Tween 80 as an emulsifier [28]. Another study showed that 20% of curcumin encapsulated in silica nanoparticle-stabilized Pickering emulsion was degraded within 20 h of storage [29]. A further study showed that approximately 14% of the encapsulated curcumin in Pickering emulsion stabilized by chitosan-tripolyphosphate nanoparticles was degraded after 24 h [30]. In addition to Pickering emulsion, about 40% of the encapsulated curcumin in liposomes was degraded in less than 200 min [31]. Compared with these reported studies, the results of this study indicated that the encapsulation of curcumin in the present O/W emulsions significantly improved the storage stability of curcumin compared to curcumin encapsulated in nano-emulsions, Pickering emulsions, and liposomes.

### 2.5. Stability of Curcumin and the O/W Emulsion during In Vitro Gastrointestinal Digestion

**Figure 11:**
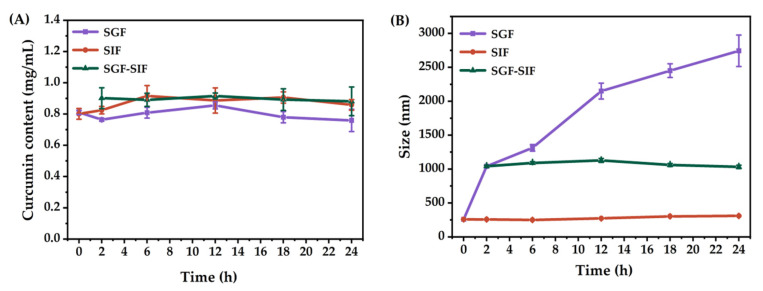
The variation of curcumin content (**A**) and mean droplet size (**B**) during in vitro gastrointestinal digestion for 24 h.

Figure 11 shows the curcumin content and the droplet size distribution of the O/W emulsion after incubation with simulated gastrointestinal fluids for 24 h. The results reveal negligible changes in curcumin content during incubation with SGF, SIF, and mixed SGF-SIF. This indicates that effective protection of curcumin was achieved by encapsulation in the O/W emulsion, even under the condition of in vitro gastrointestinal digestion. Therefore, curcumin is expected to achieve high bioaccessibility and bioavailability when the O/W emulsion is used in clinical applications.

In general, ingested food stays in the stomach for about 2 h in the gastrointestinal digestion process, then passes into the small intestine. When incubated with SGF for 2 h, the mean droplet size of the O/W emulsion increased from 260 nm to 1041 nm. After that, the digested fluid was incubated with SIF for another 22 h, and the particle size remained unchanged. For comparison, the O/W emulsion was incubated with SGF and SIF for 24 h, respectively. The mean droplet size of the O/W emulsion was unchanged during incubation with SIF. While, when incubated with SGF, the mean droplet size continuously increased to 2743 nm after 24 h, suggesting that the emulsion was destabilized during simulated gastric digestion. These results are consistent with previous findings on the influence of pH on the stability of curcumin O/W emulsions (Section 2.3).

## 3. Materials and Methods

### 3.1. Materials

Curcumin was purchased from Sigma-Aldrich (Shanghai) Trading Co., Ltd. (Shanghai, China) Triglycerol monolaurate was provided by Jinan Dowin Chemical Technology Co., Ltd. (Jinan, China). Polyoxyethylene sorbitan monooleate (Tween 80) and lecithin were obtained from J&K Scientific Ltd. (Beijing, China). Food-grade olive oil (Andalusia extra virgin) and linseed oil (Chu Cui) were obtained from COFCO Corporation (Beijing, China). Medium-chain triglycerides (MCT, batch number IOI00001) were obtained from Beijing Innochem Science & Technology co., Ltd. (Beijing, China). All the chemicals were used as received without further purification unless otherwise stated.

Simulated gastric fluid (SGF) was prepared by dissolving NaCl (2.0 g) and HCl (7 mL) in deionized water; more deionized water was then added to make the final volume up to 1 L. The pH of the final solution was tested and adjusted to 1.2 using 0.2 mol/L NaOH or 0.2 mol/L HCl solution. Simulated intestinal fluid (SIF) was prepared by dissolving KH_2_PO_4_ (6.8 g) in 500 mL deionized water, followed by adding 0.9 g NaOH. The well-mixed solution was then topped up with deionized water to 1 L, and the pH was tested and adjusted to 6.8 using 0.2 mol/L NaOH or 0.2 mol/L HCl solution.

### 3.2. Preparation of Curcumin O/W Emulsions

The curcumin solution was prepared by dispersing the dried powders in the oil phase (olive oil, linseed oil, or MCT) and stirring until completely dissolved. The aqueous solutions of triglycerol monolaurate were prepared at a concentration of 0.6%, 0.8%, 1.0%, 1.2%, or 1.4% (*w*/*w*). The oil and aqueous phases (2:8, 3:7, 4:6, or 5:5, *v*/*v*) were premixed with a high-speed homogenizer (T25, IKA Works Inc., Staufen, Germany) equipped with an S25 N-10G rotor operated at 13000 rpm for 5 min. The coarse emulsions were finely dispersed with a high-pressure homogenizer (Panda plus, Niro Soavi, Italy) with pressures of 60, 80, or 100 MPa for 2, 4, 6, 8, or 10 cycles.

### 3.3. Experimental Design for Optimizing the Preparation of Curcumin O/W Emulsions by RSM

Based on the results of single-factor experiments, four factors (concentration of triglycerol monolaurate, oil phase fraction, homogenization pressure, and number of processing cycles) were selected for multivariate optimization. The values for low, middle, and high levels of each variable were −1, 0, and 1, respectively, as shown in Table 3. 

### 3.4. Stability of Curcumin O/W Emulsions

The stability of the O/W emulsions was determined by multiangle light scattering using a Turbiscan Tower Stability Analyzer (Formulation, Toulouse, France). The emulsion was sampled immediately after preparation and placed in a cylindrical glass cell, which was scanned by a light beam emitted in the near-infrared (880 nm).

The stability of the emulsion can be determined from the uniformity of the light scattering at different depths with a wavelength of 880 nm. TSI was taken as a measure of the stability of the emulsion and can be calculated as follows:

(2) $$\mathrm{TSI}=\sum_{i}\frac{\sum_{h}\left|Scan_{i}\left(h\right)-Scan_{i-1}\left(h\right)\right|}{H}$$

where *H* is the sample height from the bottom of the cell to the meniscus, *Scan_i_*(*h*) is the *_i_*(*h*) scan at a given height *h*, *Scan_i_*_−1_(*h*) is the *_i_*_−1_(*h*) scan at a given height *h*, and *i* is a number from 1 to *k* (*k* = total time/scan speed).

### 3.5. Determination of the Droplet Size Distribution of Curcumin O/W Emulsions

The droplet size distribution of the O/W emulsions was determined using a dynamic light scattering (DLS) instrument (Zetasizer Nano S, Malvern, UK). Samples were filtrated (0.22 μm) before measurement. For each measurement, 0.5 mL of O/W emulsion was added to a polystyrene micro-UV cuvette, and DLS was measured at an angle of 90°. Each sample was measured in triplicate and reported as a calculated mean and standard deviation.

### 3.6. Quantification of Curcumin Encapsulated in O/W Emulsions

The curcumin encapsulated in O/W emulsions was quantified using a spectrophotometric method, based on the reported literature with minor modifications [22,32,33]. Firstly, the colloidal system was disrupted by diluting with ethanol (100 times). The concentration of curcumin was determined by measuring the absorbance of the diluent at 425 nm using a UV-Vis spectrophotometer (UV-3600, Shimadzu, Tokyo, Japan) at room temperature. The absorbance was then converted to curcumin concentration using a standard curve: y = 0.1749x + 0.0169 (R^2^ = 0.99975), and 0.9 µg/mL, 1.5 µg/mL, 3 µg/mL, 6 µg/mL, 12 µg/mL, and 18 µg/mL of standard curcumin solutions were used. All measurements were performed three times and were reported as calculated means and standard deviations.

### 3.7. Simulated In Vitro Gastrointestinal Digestion

Curcumin O/W emulsion (5 mL) and simulated gastrointestinal fluid (5 mL) were placed in a clean tube, followed by incubation in a shaking water bath at 37 °C and 120 rpm under dark conditions. A 100 µL sample was taken and replaced with 100 µL of the respective fresh simulated gastrointestinal fluids at a particular time point. The curcumin content of each sample was determined by UV-Vis spectrophotometry according to the method in Section 3.5. Three sets of in vitro gastrointestinal digestion experiments were conducted, curcumin O/W emulsion incubated with SGF for 24 h (SGF), curcumin O/W emulsion incubated with SIF for 24 h (SIF), and curcumin O/W emulsion first incubated with SGF for 2 h then SIF for 22 h (SGF-SIF).

### 3.8. Transmission Electron Microscopy (TEM) Observations

The morphology of the curcumin O/W emulsions was studied using a JEOL JEM-2100 transmission electron microscope at an operating voltage of 120 kV. One drop of curcumin O/W emulsion was placed on a carbon-coated copper grid (200 mesh). The grids were dried overnight and then imaged. 

## 4. Conclusions

In this study, the effects of emulsifier type, emulsifier concentration, oil type, oil-to-water ratio, homogenization pressure, and the number of processing cycles on the preparation of curcumin-encapsulated O/W emulsions were investigated. The optimized conditions were found to be: triglyceride monolaurate as an emulsifier with a mass concentration of 1.23%, MCT as the oil phase with an oil fraction of 28.1%, under 85.0 MPa of homogenization pressure, and with eight processing cycles. The optimized O/W emulsion exhibited excellent storage stability, which was confirmed by only very slight variations in droplet size during 60-day storage. In addition, a desirable physical stability of curcumin encapsulated in O/W emulsions after 60 days of storage under both natural light and dark conditions was obtained, with retention of 97.9% and 81.6% of the initial content, respectively. The mean droplet size of the O/W emulsion continuously increased from 260 nm to 2743 nm during incubation in SGF for 24 h, while remaining unchanged in SIF. Negligible changes in curcumin content were displayed during incubation with simulated gastrointestinal fluids, indicating that effective protection of curcumin was achieved by encapsulation in the O/W emulsion. In summary, O/W emulsions stabilized by triglyceride monolaurate are optimized to be the preferred choice for the encapsulation of curcumin.

## Figures and Tables

**Table 1:** Box–Behnken design for the experiments.

Run	X_1_	X_2_	X_3_	X_4_	TSI
1	0	−1	1	0	0.80
2	−1	0	0	−1	1.02
3	0	−1	−1	0	0.92
4	0	0	−1	−1	0.95
5	1	1	0	0	0.99
6	0	0	0	0	0.78
7	1	0	−1	0	0.97
8	0	−1	0	1	0.96
9	0	1	1	0	0.94
10	0	0	−1	1	0.92
11	0	−1	0	−1	0.85
12	0	1	0	1	0.89
13	−1	−1	0	0	1.03
14	0	0	1	−1	0.94
15	1	−1	0	0	0.92
16	0	0	0	0	0.74
17	1	0	0	−1	0.96
18	−1	0	−1	0	1.04
19	0	0	0	0	0.73
20	−1	0	0	1	1.02
21	−1	1	0	0	1.05
22	1	0	0	1	0.97
23	0	1	−1	0	0.89
24	0	0	1	1	0.84
25	0	0	0	0	0.72
26	1	0	1	0	0.88
27	0	1	0	−1	0.97
28	−1	0	1	0	1.01
29	0	0	0	0	0.75

**Table 2:** The results of ANOVA for Response Surface Reduced Quadratic Model.

Source	Sum of Squares	df	Mean Square	F-Value	*p*-Value	
Model	0.2644	14	0.0189	30.53	<0.0001	significant
X_1_	0.0192	1	0.0192	31.03	<0.0001	
X_2_	0.0052	1	0.0052	8.42	0.0116	
X_3_	0.0065	1	0.0065	10.56	0.0058	
X_4_	0.0007	1	0.0007	1.09	0.3139	
X_1_X_2_	0.0006	1	0.0006	1.01	0.3319	
X_1_X_3_	0.0009	1	0.0009	1.45	0.2478	
X_1_X_4_	0.0000	1	0.0000	0.0404	0.8436	
X_2_X_3_	0.0072	1	0.0072	11.68	0.0042	
X_2_X_4_	0.0090	1	0.0090	14.59	0.0019	
X_3_X_4_	0.0012	1	0.0012	1.98	0.1812	
X_1_²	0.1732	1	0.1732	279.98	<0.0001	
X_2_²	0.0438	1	0.0438	70.78	<0.0001	
X_3_²	0.0304	1	0.0304	49.07	<0.0001	
X_4_²	0.0551	1	0.0551	89.06	<0.0001	
Residual	0.0087	14	0.0006			
Lack of Fit	0.0065	10	0.0007	1.23	0.4536	not significant
Pure Error	0.0021	4	0.0005			
Cor Total	0.2731	28				

**Table 3:** The main factors and the corresponding levels.

Parameters	Factor Code	Level of Factors		
		−1	0	1
Concentration of triglycerol monolaurate (wt%)	X_1_	1.0	1.2	1.4
Oil phase fraction (%)	X_2_	20	30	40
Homogenization pressure (MPa)	X_3_	60	80	100
Processing cycles (number)	X_4_	6	8	10

## Data Availability

Not applicable.
